# StatsDB: platform-agnostic storage and understanding of next generation sequencing run metrics

**DOI:** 10.12688/f1000research.2-248.v2

**Published:** 2014-02-19

**Authors:** Ricardo H. Ramirez-Gonzalez, Richard M. Leggett, Darren Waite, Anil Thanki, Nizar Drou, Mario Caccamo, Robert Davey

**Affiliations:** 1 The Genome Analysis Centre, Norwich Research Park, Norwich, NR4 7UH, UK

## Abstract

Modern sequencing platforms generate enormous quantities of data in ever-decreasing amounts of time. Additionally, techniques such as multiplex sequencing allow one run to contain hundreds of different samples. With such data comes a significant challenge to understand its quality and to understand how the quality and yield are changing across instruments and over time. As well as the desire to understand historical data, sequencing centres often have a duty to provide clear summaries of individual run performance to collaborators or customers. We present StatsDB, an open-source software package for storage and analysis of next generation sequencing run metrics. The system has been designed for incorporation into a primary analysis pipeline, either at the programmatic level or via integration into existing user interfaces. Statistics are stored in an SQL database and APIs provide the ability to store and access the data while abstracting the underlying database design. This abstraction allows simpler, wider querying across multiple fields than is possible by the manual steps and calculation required to dissect individual reports, e.g. ”provide metrics about nucleotide bias in libraries using adaptor barcode X, across all runs on sequencer A, within the last month”. The software is supplied with modules for storage of statistics from FastQC, a commonly used tool for analysis of sequence reads, but the open nature of the database schema means it can be easily adapted to other tools. Currently at The Genome Analysis Centre (TGAC), reports are accessed through our LIMS system or through a standalone GUI tool, but the API and supplied examples make it easy to develop custom reports and to interface with other packages.

## Introduction

Next generation short-read sequencers are now capable of generating hundreds of gigabases of sequence data per run. This increase in throughput is complemented by technologies such as long-read single molecule platforms, multiplex sequencing and RADSeq ^1, 2^, that lead to differing analytical techniques and enable hundreds of samples to be combined into a single sequencing experiment, respectively. With this data heterogeneity and abundance comes a significant challenge to keep track of samples, to understand data quality and to understand how quality control (QC) and yield are changing across instruments and over time. As well as the desire to understand historical data, centres often have a duty to provide clear summaries of individual run performance to collaborators or customers.

A user’s initial attempts to understand the QC profile of a run usually involve using manufacturer-supplied software, for example Illumina’s Sequence Analysis Viewer or PacBio’s SMRTAnalysis. As well as these, a number of community-developed tools have emerged for assessing run quality and they are often included in the primary analysis pipelines of sequencing centres. FastQC ^3^ is a popular tool for analysing FASTQ files and is able to report a wide range of information related to the quality profile of the reads, as well as analysing GC content and over-represented sequence, such as PCR duplicates or over-abundance of adaptors. FastQC will output a set of HTML files and also a single plain-text flat file suitable for parsing by third party tools. HTQC, another toolkit for FASTQ data analysis, is composed of a set of six tools for analysis and trimming of reads ^4^. PRINSEQ also analyses and trims reads, with an emphasis on metagenomic datasets ^5^. Other tools include NGSQC ^6^, QRQC ^7^ and SAMStat ^8^. The latter tool, as the name implies, works with SAM files rather than FASTQ files.

The new generation of single molecule sequencing technologies, such as the RS platform from Pacific Biosciences with longer reads and different error profiles, have brought their own QC challenges. While it is still possible to get useful information from next generation tools such as FastQC, new tools are emerging which are tailored to the platform. Examples include stsPlots, which provides graphical summaries of data included in the sts.csv files output by the instrument ^9^ and PacBioEDA which operates on the .bas.h5 files produced by the PacBio primary analysis ^10^.

While there are a range of useful tools available to generate QC statistics for individual runs, we are not aware of any currently available solution for facilitating the easy storage and access of this valuable information. This tends to lead to many flat files stored on disc in multiple locations and a lack of coherent analysis. For this reason, we have created StatsDB, a platform-independent, tool-independent run QC and metadata database with APIs in Perl and Java. StatsDB features a generic database schema which enables the storage of data from any QC tool designed for any sequencing platform. StatsDB is designed to automate the storage of run QC metrics, enabling more granular queries over the data held within. Installation is simple and use of the software and API requires no knowledge of SQL.

## Methods and implementation

**Figure 1.**
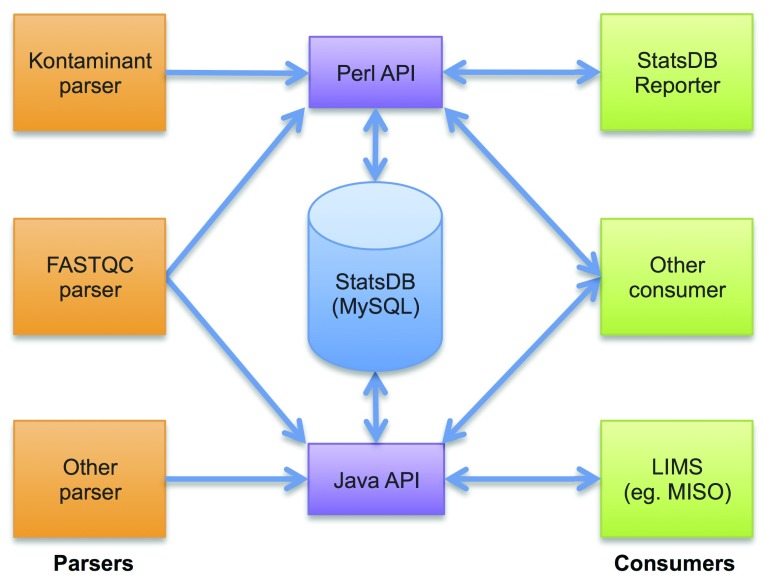
At the core of StatsDB is a MySQL database supported by Perl and Java APIs. Parsers are simple scripts to write the output of QC tools to the database, while Consumers present the database contents to users.

Figure 1 illustrates the overall structure of the StatsDB system. At the core of StatsDB is a MySQL database which stores run metrics on a per-base, per-partition or per-run basis. On top of the database sits an API - currently implemented in both Perl and Java - which abstracts the database design from the tools that use the data and provides a simple interface for adding or querying data.

We envisage two types of programs utilising the API ­ *parsers* and *consumers*. Parsers process external data out­put from QC tools and use the StatsDB API to write the data. Conversely, consumers query the data stored in StatsDB and present it to the user, typically in the form of text and graphs. The StatsDB package includes parser implementations and examples of consumers, and the API enables others to be developed quickly and easily. Additionally, integrating these consumers into third-party applications is promoted - the open source LIMS system currently in development at TGAC, MISO ^11^, provides support for accessing data in StatsDB out-of-the-box.

### Database installation

The StatsDB framework supplies two SQL files that are used to set up the database for use, comprising the schema and the stored procedures, respectively, and detailed in the following sections.

The first step is to create a new MySQL database called ‘statsdb’ and grant a user read-write access to it, e.g. a new ’statsdb’ user with a suitable password, e.g. 'statsdb’:



                        mysql > CREATE DATABASE statsdb;
mysql > USE statsdb;
mysql > GRANT ALL ON ‘statsdb‘.*
              TO ’statsdb’@’localhost’;
mysql > GRANT ALL ON ‘statsdb‘.*
              TO ’statsdb’@’localhost’
              IDENTIFIED BY ’statsdb’;
                    


The two SQL files are then imported into the database as follows:



                        $ mysql -D statsdb -u statsdb -p \\
                 < statsdb_schema.sql
$ mysql -D statsdb -u statsdb -p \\
                 < stored_procedures.sql
                    


This will populate the ‘statsdb’ database with the tables and procedures required.

Testing a successfully installed database can be undertaken by running the FastQC parser on the supplied example data, as follows:



                        $ 
                        cd Perl
$ perl parse_fastqc.pl \\
       -i examples / metadata_test.txt \\
       -d examples / template_db.txt


This will result in data being inserted into the database. In order to revert back to an empty state, reimport the schema SQL as detatiled previously.

### Database design

**Figure 2.**
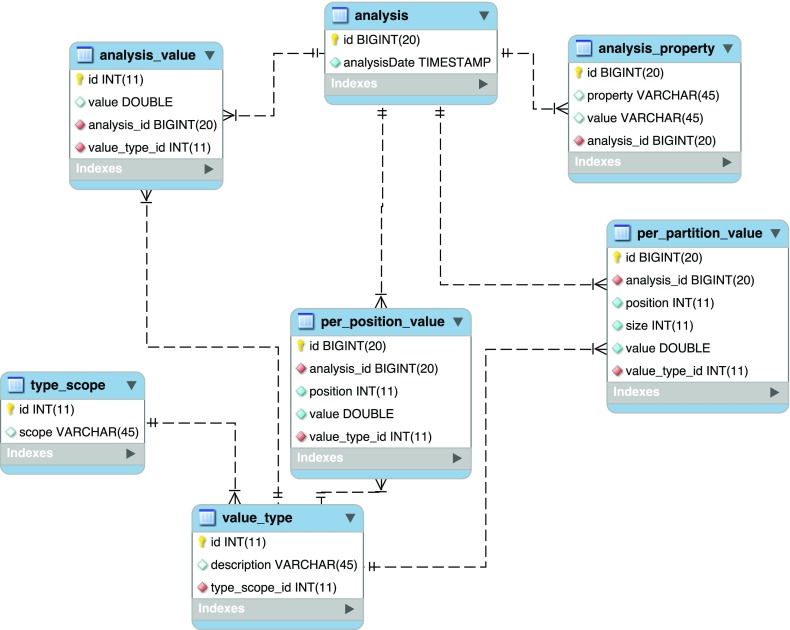
StatsDB database schema.

The StatsDB database is designed to be flexible and to hold virtually any type of QC analysis. Figure 2 illustrates the database schema. The database is normalised to the third normal form (3NF) and has stored procedures and views to facilitate consistent access to the stored information. The tables in the core of the database are as follows:

**analysis** holds the ID and timestamp of when the analysis was recorded. If an analysis is carried out with the same parameters, this constitutes a new analysis and an additional unique analysis table entry will be created.

**analysis_property** holds general information about the analysis and the run from which it originated. The values contained within this table are populated directly from a user-specified tab-delimited table of property headings and respective values, i.e. the *RunTable* object (see ’Parsers’ below). The following properties are used to define common denominators across platforms and analyses:

- **tool** - the name of the tool that was used to undertake the analysis (for example, FastQC, PRINSEQ).
- **encoding** - the encoding of the input, for example ‘Illumina 1.5’, ‘fasta+qual’.
- **chemistry** - a short name for the chemistry or type of experiment run.
- **instrument** - the ID of the instrument.
- **software** - the basecaller software or software version of the instrument.
- **type** - type of experiment, for example ‘RNA-Seq’, ‘WGS’.
- **pair** - if the experiment is paired end or mate pair, then 1 if the first read or 2 if the second read.
- **sample_name** - the name of the sample as as­signed during the library construction.
- **lane** - the lane or flowcell of the instrument.
- **run** - the name of the run, as given by the in­strument.
- **barcode** - if the sample is multiplexed, the adaptor barcode of the sample, for example ‘ACCTTG’.

**analysis_value** is used to store the properties that can be represented as a value for the whole run, e.g. over­represented sequences, counts of multiplexed tags.

**per_position_value** stores values related to a absolute position that is never grouped, such as counts of how many times a certain quality is represented.

**per_partition_value** is similar to *per_position_value*, but allows grouping by some range or factor.

**value_type** is an auxiliary table that holds descriptions for the values and the scope of the value.

**type_scope** is an auxiliary table defining the scope relevant to the analysis to group the values consistently. Currently, the following are the scopes supported by StatsDB.

- **analysis** - global values for the analysis, for example ‘Total GC content’.
- **base_partition** - values of summaries per base, allowing grouping of ranges, for example quality mean per position.
- **sequence_cumulative** - for cumulative counts, such as quality scores or read counts.

In addition to the tables, StatsDB has three auxiliary views, used to facilitate complex queries:

**property** transposes the *analysis_property* table, transforming the row values to columns.

**run** merges the corresponding values from *property*, according to the run.

**latest_run** selects the latest run from the *run* view, so if an analysis has been carried out more than once (see analysis table above), only the latest results are stored.

### Stored procedures

Most of the functionality of StatsDB is embedded directly in the database as views and stored procedures. This enables it to have lightweight APIs which behave consis­tently across languages and means that the API can easily be ported to other languages should the implementation require it.

The following stored procedures are used by the APIs to query the database:

**list_runs** will list all available runs.

**list_selectable_properties** will list the properties that can be used as queries.

**list_selectable_values_from_property** will list the values for a given property.

**list_summary_per_scope** will return the descriptions present for a given scope. This is useful to know if a *value_type* is already present or not, before trying to insert it again.

The following stored procedures are used to generate re­ports. To make queries, the common arguments to spec­ify the run or runs to group are: *instrument_in, run_in, lane_in, pair_in* and *barcode*. The arguments are op­tional and if more than one analysis meets the query criteria, a summary is produced. This can be used, for example, to query the quality of a given lane and use this information to find systematic issues.

**general_summaries_for_run** returns a summary of all the values global to the analysis.

**summary_per_position_for_run** returns all the values queried from the *per_partition_value* table.

**summary_value_with_comment** returns the summary values with a descriptive text, if it was present as a note for a value. For example, this can be used to retrieve further description of the over-represented sequences in a FastQC report.

### API

The StatsDB framework provides two APIs, one in the Perl language and the other in Java, both of which offer the same functionality. They call the stored procedures and provide a sufficient layer of abstraction such that parsers and consumers do not need to access the stored procedures directly. Therefore, the typical method of accessing the data held within StatsDB is through the following APIs.

### Perl API

Before using the Perl API, a connection to the database has to be created as defined by the Perl DBI API ^12^. A template for the configuration file is provided in *Perl/examples/template_db.txt*, and the values need to match those used in the database installation instruc­tions above:



                        db_string    dbi:mysql:statsdb;host=localhost
db_user      statsdb
db_password  statsdb
                    


The Perl API comprises modules to import analysis information to the StatsDB database, as well as functions to query the database. The abstraction of the database is contained in the *QCAnalysis* module, which is used to add an analysis to the database. It automatically fills the missing types of value in the database, so it is not necessary to have a comprehensive list of types of analysis that are going to be stored a *priori*. However, a parser needs to define valid types for the *QCAnalysis* object, i.e. *value_type*, and to which scope they should be assigned, i.e. the *type_scope*, as described in the Database design section above, and in the following code:



                        // function signature
$analysis->add_valid_type ($value_type,
                           $value_scope);
// examples
$analysis->add_valid_type (
                        "general_gc_content",
                           
                        "analysis");
$analysis->add_valid_type (
                        "gc_content_percentage",
                           
                        "base_partition");
$analysis->add_valid_type (
                        "gc_content_count",
                           
                        "sequence_cumulative");
$analysis->add_valid_type (
                        "base_content_c",
                           
                        "base_partition");


After defining the valid values and scopes, the properties of the analysis should be added. The following example adds a 'tool’ property with a 'FastQC’ value, to represent a FastQC analysis property type:



                        $analysis->add_property  (
                        "tool",
                        "FastQC");


Global values are supported in StatsDB to represent generalised properties that are permissible across analyses. To add global values to the analysis, the *add_general_value* function is used. General values can have an optional description. When the description is present, and if the value is new to the database, the description is added. If the value is already present in the database, the description is ignored. This is by design and allows for consistency across analyses:



                        // function signature
$analysis->add_general_value ($key,
                              $value,
                              $description);
// examples
$analysis->add_general_value (
  
                        "ACCTGATAT",
  10,
  
                        "over-represented⌴common⌴primer⌴in⌴library⌴A"
);
$analysis->add_general_value (
  
                        "average_length",
  100
);


To add values with a discrete count the *add_position_value* function is called. The following example specifies that 15,000 reads in the analysis had a quality score of 30:



                        // function signature
$analysis->add_position_value ($position,
                               $key,
                               $value);
$analysis->add_position_value(
  30,
  
                        "quality_score_count",
  15000
);


Finally, to add values that can be grouped in ranges the function *add_partition_value* is called. A range is an array specifying the first and last position (inclusive). The following example inserts a quality mean from position 10 to position 14 (5 values) of the run:



                        // function signature
$analysis->add_partition_value ($range,
                                $key,
                                $value);
$analysis->add_partition_value (
  [10-14],
  
                        "quality_mean",
  38.7
);


An auxiliary function, *parse_range*, is provided to convert a string representation of a range into an array type. If a single string value is provided, it returns an array with the value repeated, representing a partition of size 1. If a range string is passed, it returns an array with the positions as needed by *add_partition_value*:



                        $analysis->parse_range {
                        "10"} -> [10,10]
$analysis->parse_range {
                        "10-14"} -> [10,14]


Once the *QCAnalysis* object is constructed with all the required values, it can be inserted to the database with *db- > insert_analysis*$ *(analysis)*.

To query the database from the Perl API, the *Reports.pm* module is used. To allow flexibility in the querying and to be able to get summaries at different granularities (bar­code, lane, pair, etc) the convention is that all the queries accept as an argument a properties hash comprising the required key-value pairs to build the query. A constant, declared in the *Reports.pm* module, represents the set of controlled platform-agnostic keys and is defined as follows:



                        
                        use constant {
 ENCODING => 
                        "encoding",
 CHEMISTRY => 
                        "chemistry",
 INSTRUMENT => 
                        "instrument",
 SOFTWARE_ON_INSTRUMENT => 
                        "softwareOnInstrument"
   ,
 TYPE_OF_EXPERIMENT => 
                        "typeOfExperiment",
 PAIR => 
                        "pair",
 SAMPLE_NAME => 
                        "sampleName",
 LANE => 
                        "lane",
 BARCODE => 
                        "barcode",
 RUN => 
                        "run"
};


An example script to query the different types of tables is provided with the StatsDB framework, i.e. *examples/example_consumer.pl*.

### Java API

The Java API is supplied as a Maven ^13^ project to ease building and testing. For convenience, a pre-built JAR file is available to use in existing Java projects by simply downloading the JAR file from the TGAC Maven repository ^14^, or including the following repository and dependency Maven declarations in your pom.xml build descriptor to download the artifact:



                        <repository>
 <id>tgac-repo</id>
 <name>TGAC Maven Repository</name>
 <url>https://repos.tgac.ac.uk/maven/repo</url>
</repository>
...
<dependency>
 <groupId>uk.ac.tgac.statsdb</groupId>
 <artifactId>statsdb-api</artifactId>
 <version>1.1</version>
</dependency>
                    


The API is built using the standard Maven command:



                        mvn clean install
                    


This will compile the source code and provide a library JAR comprising the API, but does not attempt the StatsDB database-level tests. These unit tests make sure the database is accessible, that the schema is correct, and that the API calls available work cor­rectly. To turn these tests on, supply the rele­vant database connection properties in *Java/statsdb – api/src/test/resources/test.statsdb.properties*:



                        statsdb.driver=com.mysql.jdbc.Driver
statsdb.url=jdbc:mysql://localhost:3306/statsdb
statsdb.username=statsdb
statsdb.password=statsdb
                    


Then use the following profile activation when building the library:



                        mvn clean install -DdbTests=
                        true
                    


An option to build an executable JAR file is available which includes a dedicated command-line application that allows API access for loading and querying a StatsDB database. To enable this option, use the following build command:



                        mvn clean install -Donejar=
                        true
                    


The resulting JAR can then be executed by the user. This helper application requires either an input file rep­resenting the analysis report to be parsed, e.g. a *fastqc_data.txt* file, or a StatsDB metadata table file (see Table 1) comprising multiple analysis reports. The helper application will then process the analysis file(s) and load the data into StatsDB. Supplying the – *t* option allows testing of a given parser without writing any information into the database.



                        $ java -jar statsdb-api.one-jar.jar -h
usage: statsdb.one-jar.jar
 -f <file>         Use given input report file
 -h                Print this help
 -m <file>         Process multiple reports
                   using a StatsDB metadata
                   table file
 -p <fastqc,other> Use specified parser type
 -r <run>          Associate the report with
                   a given run name
 -t                Test mode. Doesn’t write
                   anything to the database
 -v                Verbose mode. Use 
                        if you
                   like lots of tasty output
 INFO [main] –  No parser type specified. Using 
                FASTQC as the default report type
ERROR [main] –  No input metadata or report 
                file specified.


**Table 1.** Example fields in an analysis metadata table file.

Field	Example
TYPE_OF_EXPERIMENT	NGS
PATH_TO_ANALYSIS	/path/to/fastqc_data.txt
ANALYSIS_TYPE	FastQC
INSTRUMENT	MISEQ-1
CHEMISTRY_VERSION	TRUSEQ_SBS_V3
SOFTWARE_ON_INSTRUMENT_VERSION	MCS_2.2.0_RTA_1.17.28.0
CASAVA_VERSION	1.8.2
RUN_FOLDER	/path/to/run_folder
SAMPLE_NAME	TEST_SAMPLE
LANE	1
BARCODE	AAACTGA
PAIR	1
RUN	RUN_NAME

Before using the Java API library, a connection to the database has to be created in a similar way to the Perl API. This file needs to be called *statsdb.properties*, needs to reside on the classpath (in the case of the executable JAR, this would simply be in the same directory, for example), and contains the same fields as the test properties example above. Finally, as with the Perl API, the values need to match those used in the database installation instructions above.

In terms of building and initiating queries within cus­tom applications, the Java API mirrors the Perl API whereby the abstraction of the database is contained in the *QCAnalysis* class. Similarly, the *Reports* class is used to query the database from the Java API, and the query key granularity is represented as a *Map < RunProperty, String >* comprising the required key-value pairs to build the query. For convenience, the API tolerates missing values and, in such a case, the aver­age is returned by default. When explicitly specifying these values, in the same way as the Perl API constants, the *RunProperty* enumeration represents the set of controlled platform-agnostic keys.

The following is a minimal example to query each of the different types of tables. Note that the first step is to construct a map of the values to query. In this particular example, the barcode is explicitly specified, i.e. "ACCGTT". If this was to be omitted, a general summary for lane 1 would be returned instead of a specific sample, allowing for an overview assessment of the run:



                        
                        // Setup the query arguments
Map <RunProperty, String> properties
  =  
                        new HashMap<>();
properties.put (RunProperty.lane, 
                        "1");
properties.put (RunProperty.barcode, 
                        "ACCGTT");
properties.put (RunProperty.run, 
                        "RUN-123");


                        // Get summary values of the run
ReportTable table = r.getAverageValues (
  properties
);
log.info (table.toJSON());


                        // Get the quality mean of the selected run across
      partitions
table = r.getPerPartitionValues (
  
                        "quality_mean",
  properties
);
log.info (table.toCSV());


                        // Get the quality score count per base position
table = r.getPerPositionValues (
 
                        "quality_score_count",
 properties
);
log.info (table.toCSV());


All javadoc for the StatsDB Java API can be found at https://repos.tgac.ac.uk/statsdb/javadoc/latest/.

### Parsers

StatsDB parsers are small programs, usually scripts, which take the output of a QC tool and use one of the APIs to store the data. Parsers for StatsDB should have the same structure, e.g. adhering to the contractual Java interfaces, and only ever need implement the specific parsing code for the analysis to be added.

Both parsing APIs require analyses to be defined in an analysis metadata table (see Table 1). This is represented as a simple tab-delimited flat file describing the list of analysis fields and values. This file should supply one analysis per line, for example a path to a FastQC data file and related properties.

**Figure 3.**
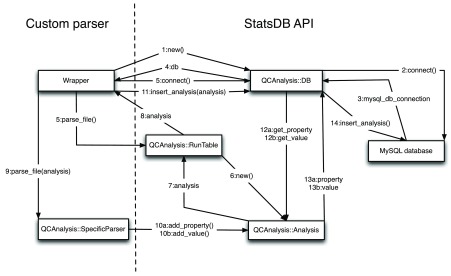
StatsDB parser interaction diagram, using the Perl API. To add new parsers, only the *SpecificParser* has to be written. The rest of the classes abstract the interaction with the database and ensure the consistency of the data. The Java API utilises different class and method names but employs the same generalised interactions.

Every new parser should conform to the contract shown in Figure 3. To write a parser it is not necessary to know the schema of the database or to modify the contents directly. StatsDB conveniently provides the database ac­cess objects within its APIs which then connect to the database. The *Wrapper* object represents a managerial entity that calls a *SpecificParser* to actually undertake the parsing, but also manages connections to the underly­ing StatsDB DB object. The *Wrapper* should open a con­nection to the database, then create a *RunTable* object to contain a list of runs, its properties and a path to the analysis to be stored, as described by the metadata table. The path is then used on the call to the *SpecificParser*, an object that creates an *Analysis* object with the properties from the run and the values in the file with the analysis. The *Analysis* object holds the values in the categories described above. Once the parsing of the file is complete, the parser forwards the *Analysis* object to the DB object, which inserts the properties and values of the analysis. Finally, the *Wrapper* object should close the connection in the DB object.

An example *Wrapper* application would be implemented in Perl as follows:



                        
                        # Opens a connection to the database

                        my $db = QCAnalysis::DB->new();
$db->
                        connect ($config);


                        # Reads the metadata table file (Figure 3)

                        my @analysis = QCAnalysis::RunTable->parse_file (
    $input);

                        # Iterates over each analysis to add

                        foreach (@analysis){
  
                        # Gets the path to the file
  
                        # The argument is the column name
  
                        my $fast_qc_file = $_->get_property (
                        "
      path_to_counts");
   
 
                        # Executes specific TagCount parser
 QCAnalysis::TagCount->parse_file ($fast_qc_file,
      $_);
      
 
                        # Inserts the analysis to the database
 $db->insert_analysis ($_);
}

                        # Closes the connection to the database.
$db->disconnect ();


A concrete *Wrapper* and *SpecificParser* implementation that interrogates FastQC output can be found in *Perl/parse_fastqc.pl* and *Perl/QCAnalysis/FastQC.pm* in the Github repository. Similarly, the Java version of a *SpecificParser* can be found in *FastQCReportParser.java*. Examples of wrapper construction of the parser and analysis objects can be seen in the *StatsDBApp.java* and *TestFastQCParser.java* classes.

### Consumers

Consumers are programs that process StatsDB data through the API. StatsDB provides a comprehensive API to query for summaries or results of an analysis related to a specific run. The client needs to know if the value is global to the run, per position, or per partition in order to select the relevant method to call. To provide a consistent interface, all the queries are summaries and the following properties can be used as selecting criteria: *encoding, chemistry, instrument, softwareOnInstrument, typeOf Experiment, pair, sampleName, lane, barcode, run*. If all the properties are specified, only the latest corresponding analysis for the run is returned. This approach allows consumers to make complex comparative analyses.

The Perl API *examples/example_consumer.pl* script provides example calls to the *Reports.pm* reporting module, which forms the basis for any consumer implementation. Similarly, to facilitate these complex queries in the Java API, we provide a *ReportsDecorator* class which contains specific methods to interact with each report, but also encapsulates related reports. One example is *getPerPositionBaseContent()* which produces a matrix of the base content of each base per position, rather than producing individual queries for each base.

**Figure 4.**
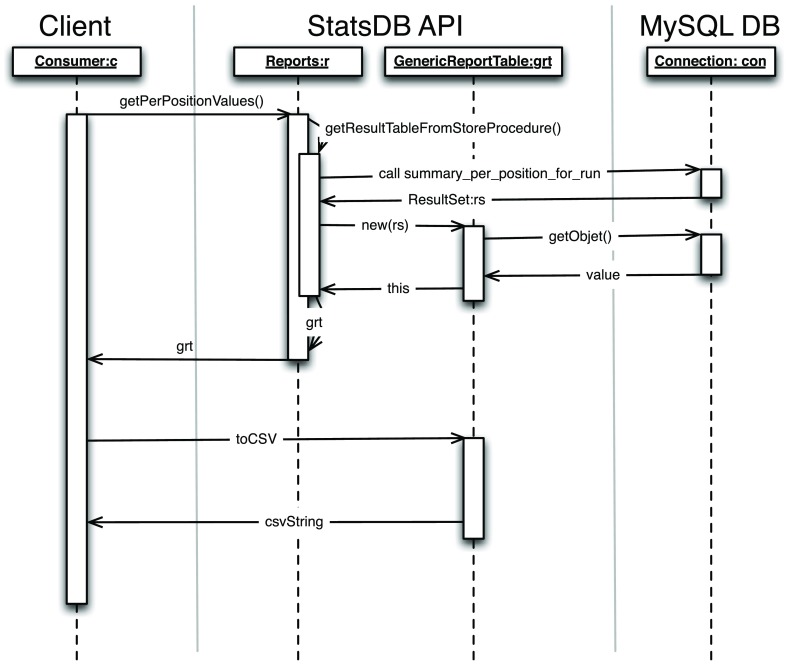
Example of the execution of a query to the database. The client only needs to be aware of the Java/Perl API and StatsDB will format the result in CSV or JSON, so that the client can display the summary.

Figure 4 shows the general approach to query the database. The consumers call the corresponding summary or the report decorator. The queried properties are then used to call the stored procedures. StatsDB reads the result set to produce an internal representation of the table, which can then be used directly by the consumer, or can be formatted as CSV or JSON. For further details and the available methods, the inline perldoc or javadoc documentation provides a comprehensive description of each method.

**Figure 5.**
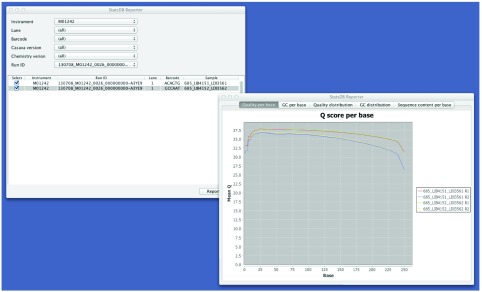
StatsDB Reporter allows overlay plots of QC data across instruments and runs.

**Figure 6.**
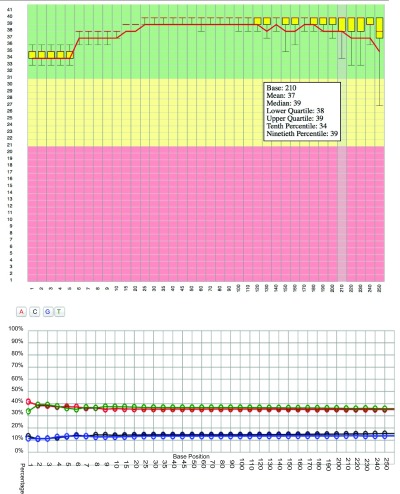
Examples of d3.js plots, generated from FastQC data parsed into StatsDB.

Reporting tools such as FastQC produce informative plots to visualise run metrics. StatsDB supplies example plots based on those produced by FastQC, written using the d3.js Javascript library ^15^, to demonstrate downstream processing and representation of consumer-generated StatsDB JSON. In this way, developers can easily integrate StatsDB plots on their own web pages. A dedicated helper class to generate a set of required JSON fragments is supplied, i.e. *D3PlotConsumer.java*, and downstream Javascript and HTML that renders the JSON fragments can also be found in the GitHub repository in the *Web* directory. Examples of interactive plots generated from FastQC per-base quality and per-base GC content can be seen in Figure 6.

## Use case

At TGAC, we use StatsDB as part of our Primary Analysis Pipeline ^16^. Each Illumina run sequenced at TGAC, both with HiSeq and MiSeq instruments, passes through this pipeline. Two important steps in the process are QC analysis with FastQC and contamination analysis using an in-house kmer-based screening tool. The output of both of these tools is parsed using two separate Perl scripts provided as part of the StatsDB package, and loaded into StatsDB. As well as these QC output, we load details of the instrument, chemistry version, RTA version and Casava version into StatsDB.

Our PacBio primary analysis pipeline is still under development, but this currently includes using FastQC to analyse FASTQ files output as part of the process. We are also working on parsers for the sts.csv files that are produced by the instrument. With StatsDB, it is perfectly possible to mix data from different platforms and different tools into one database. When querying the data, the consumer application can make the decision about what data comparisons are meaningful.

We currently access data stored in StatsDB using two consumers: TGAC’s open-source LIMS, MISO, and the proto­type StatsDB Reporter tool. Using the MISO web-based interface, it is possible to access StatsDB information and produce graph plots of FastQC data, which are based on the d3.js consumer examples supplied with the frame­work ( Figure 6). StatsDB Reporter allows selection of runs by instrument, lane, run, sample or barcode and provides comparison of data across runs ( Figure 5).

## Conclusions

Many software tools exist for the generation of run quality statistics and FastQC is possibly the most notable example. However, until now, there has been no easy solution to the problem of storage and analysis of historical run metadata and statistics. StatsDB has been designed to address this problem and offers a flexible, easy to use, platform-agnostic and tool-independent framework for consolidated access to run metrics.

Installation of StatsDB and integration with existing analysis pipelines is achieved with minimal effort. To perform data entry into StatsDB, a parser is included for the popular FastQC tool, and parsers for other tools can be written in less than a day by a competent programmer or scripter. Similarly, command line tools are provided in both Perl and Java to load parsed data into StatsDB.

To perform downstream analysis and visualisation of data held within StatsDB, reporting helper entities are provided. We envisage that smaller labs with sequencing capability would benefit from access to simple desktop tools rather than more heavyweight implementations provided by integration with a LIMS system, which would suit a larger sequencing centre. As such, an example lightweight consumer tool is supplied in the form of the StatsDB Reporter application which currently exists in prototype form but a mature version will be available in due course from the GitHub repository for StatsDB, as well as parsers for other tools (for example, PacBio sts files).

## Software details

Homepage: http://www.tgac.ac.uk/tools-resources/Source code: https://github.com/TGAC/statsdb and 10.5281/zenodo.7534 Licence: GPL v3.
