# Supplementary material for: StatsDB: platform-agnostic storage and understanding of next generation sequencing run metrics
Source: F1000Res. 2014 Feb 19;2:248. Originally published 2013 Nov 15. [Version 2] doi: 10.12688/f1000research.2-248.v2 (PMC3938176; doi:10.12688/f1000research.2-248.v2)
Supplement: Peer review history

# Peer review: Referee response for version 2

**DOI:** 10.5256/f1000research.3781.r3790

**Published:** 2014-02-28

**Authors:** Cyriac Kandoth

**Affiliations:** 1 The Genome Institute, Washington University, St Louis, MO, USA

The revised version of this article, and the author's responses to my questions and comments, are satisfactory. The plan to stick to GPLv3 is commendable. And the FrontiersIn article has a decent figure showing a data flow with StatsDB in the pipes. I have no further requested revisions. Cheers!

I have read this submission. I believe that I have an appropriate level of expertise to confirm that it is of an acceptable scientific standard.

# Peer review: Referee response for version 2

**DOI:** 10.5256/f1000research.3781.r3788

**Published:** 2014-02-21

**Authors:** Anuj Kumar

**Affiliations:** 1 Department of Molecular, Cellular & Developmental Biology, University of Michigan, Ann Arbor, MI, USA

The revisions address my comments, and I have no further requested revisions.

I have read this submission. I believe that I have an appropriate level of expertise to confirm that it is of an acceptable scientific standard.

# Peer review: Referee response for version 1

**DOI:** 10.5256/f1000research.2894.r2794

**Published:** 2013-12-27

**Authors:** Anuj Kumar

**Affiliations:** 1 Department of Molecular, Cellular & Developmental Biology, University of Michigan, Ann Arbor, MI, USA

The manuscript by Ramirez-Gonzalez *et al.* presents a software package to store and analyze metrics from next-generation sequencing runs.  The open-source software package, StatsDB, stores statistics from sequencing reactions in a MySQL database, and APIs provide means to access and analyze the data.  The StatsDB package facilitates easy querying, particularly across multiple fields from respective tables in the database. StatsDB provides modules that can be used in conjunction with FastQC as well as with other tools suited for the analysis of sequence data.

From the perspective of a wet-lab genomicist, the StatsDB package seems to be a useful tool, and I can certainly see the utility in storing data from the sequencing reactions, particularly for a genome analysis center. I only have a few minor comments.

 

1. Since FastQC is commonly used for the analysis of sequencing runs, and since the StatsDB package works with FastQC, it would be useful to provide a little more explicit information describing the output/types of data provided by FastQC.  Some of these data are presented in the StatsDB schema, but it would be nice to add a bit more detail in this regard (possibly a very simple figure) into the Introduction.
2. I appreciate the use case provided, but I think it would be helpful to provide an additional small example of how the StatsDB package could be useful to an individual lab that uses a moderate level of next-generation sequencing in their research (as opposed to a genome center).  Possibly, a summary of the StatsDB Reporter application would suffice.
3. It would be helpful to add a few sentences to the Introduction to indicate how other sequencing centers, etc. store output from FastQC or other similar tools. This does not have to be an extensive overview.

I have read this submission. I believe that I have an appropriate level of expertise to confirm that it is of an acceptable scientific standard.

# Response

**Published:** 2014-01-20

**Authors:** Robert Davey

**Affiliations:** The Genome Analysis Centre, UK

We would like to thank Anuj for taking the time to read and comment on our manuscript.

We have added a sentence in to the Introduction, outlining briefly the data formats outputted by FASTQC, i.e. a set of HTML files, and a single plain-text flat file from which we parse the data to be loaded into StatsDB.

We envisage no differences in scaling terms between a small-scale lab with one or two sequencers and a large multi-platform centre. StatsDB has applications in the smaller centre where quick but potentially more sporadic access to historical run data would be investigated through the StatsDB Reporter tool rather than the more "heavyweight" integration with a LIMS or via a web server. As such, we have added a sentence describing its relevance in this context. We will be publishing the StatsDB Reporter application separately, or as a software update to this publication, in due course.

We have added a short overview of how the output of QC tools might be kept in different centres, highlighting the usefulness of StatsDB.

# Peer review: Referee response for version 1

**DOI:** 10.5256/f1000research.2894.r2565

**Published:** 2013-12-16

**Authors:** Cyriac Kandoth

**Affiliations:** 1 The Genome Institute, Washington University, St Louis, MO, USA

The authors have developed a tool that is well documented, open sourced, and version controlled. The first two thirds of the article read like a user's manual, which is appropriate in a technical article, but not in its entirety. The neatly organized code snippets and commentary will help a programmer who is considering using the API. However, to encourage them to use it, the **Use case** section must be expanded with figures and performance metrics. I have added more details in the comments below.

**Major comments:**

1. Title - " *platform-agnostic storage and understanding...*" - The word "understanding" seems either ambiguous or inappropriate since StatsDB is just the facilitator for interpreting/visualizing the run metrics. Consider a title like " *StatsDB: platform-agnostic storage schema and API for interpreting next-generation sequencing run metrics*"
2. Page 4, Database design - The relationship between the **analysis** table and the auxiliary views is unclear. For example, the **analysis** table stores a unique ID and timestamp of when an analysis run was performed. But the **latest_run** view mentions that an analysis can be run more than once. Please clarify.
3. Page 10, Use case - Add a figure with a flowchart showing the TGAC pipeline that is explained in the text. It is critical to show how StatsDB plugs into a standard analysis pipeline.
4. For the source code, an LGPL license might be more appropriate than GPL because StatsDB is an API, rather than a standalone tool in a workflow. LGPL would also allow parsers/consumers for manufacturer-supplied metrics.

**Minor comments:**

1. Page 3, Database installation - The code snippets show file names with accidental whitespace. Specifically: *statsdb_schema.sql, stored_procedures.sql, and parse_fastqc.pl*
2. Page 3, Introduction - " *With this data heterogeneity and abundance, comes a..*." - missing comma.
3. Page 5, Database design - How would you store metrics that depend on genomic positions or ranges? e.g targeted sequencing coverage. If tables **per_position_value** and **per_partition_value** stored values based on genomic positions or ranges, it would also depend on which reference sequence build (e.g. NCBI36, GRCh37) was in use for that analysis. Would we define that build as a **value_type**? And in the stored procedures used by the APIs, how can we handle a mix of different reference builds used across runs?
4. Page 7, Java API - accidental whitespace in filename *fastqc_data.txt*

I have read this submission. I believe that I have an appropriate level of expertise to confirm that it is of an acceptable scientific standard, however I have significant reservations, as outlined above.

# Response

**Published:** 2014-01-24

**Authors:** Robert Davey

**Affiliations:** The Genome Analysis Centre, UK

We would like to thank Cyriac for taking the time to read and comment on our manuscript.

In terms of the Use Case expansion, we believe the usefulness is inherent in the ability to store, retrieve and therefore compare historical run metrics, based on a variety of user- or analysis-specific attributes. We envisage tools like StatsDB Reporter will be the simplest and more widely-used interface with StatsDB for the average lab technician or bioinformatician. Similarly, we believe performance metrics at this level would be unhelpful rather than beneficial, as these would be very dependent on infrastructure, hardware and DMBS used. We aim to publish incremental updates to the API and surrounding tools, e.g. a full StatsDB Reporter release, and a Python API.

The StatsDB schema was designed to allow the same analysis to be stored multiple times, with potentially differing or identical parameters. We foresee that if analysis uniqueness is required, this would be down to an API implementation to check for previous analyses with the same tool and parameters supplied, rather than at the database level. We have added a description to the Database Design section (analysis and latest_run table outlines) of the manuscript.

The pipeline that utilises StatsDB has been covered in detail in our recent open-access FrontiersIn publication, so we have added a reference to this paper in the Use Case main text.

The GPLv3 aims to give free software developers an advantage over proprietary developers. A parser that supports a proprietary format does not fit within the scope of our vision - we aim to continue the trend in bioinformatics software whereby fully open-source licences are preferred to maximise the reusability of a given tool or library.

Yes, you are correct in using value_type for this kind of attribute. There are API calls that let you pull out analyses by a value_type value, so this should be supported out of the box.

The accidental whitespace has been introduced in the HTML view, from which the PDF download is generated. We shall contact the editorial office to ensure these are corrected. Thank you for spotting those!

# Peer review: Referee response for version 1

**DOI:** 10.5256/f1000research.2894.r2467

**Published:** 2013-12-10

**Authors:** Mick Watson

**Affiliations:** 1 ARK-Genomics, University of Edinburgh, Edinburgh, UK

The article is well written and describes a tool which will be very useful to the community.

I only have a few minor issues:

1. Throughout I felt that the terms used were confusing: "analysis" and "run" for example. These can take on multiple meanings in sequencing (for example, what the SRA calls a run is not what I call a run). I feel these terms need a better definition when they are first introduced, with examples where appropriate.
2. Is it valid to still use the term "short-read" sequencers?
3. There is a lot of technical information about the design, database and APIs, but only a single example (using Illumina data) at the end; examples using PacBio and Ion Torrent data would show the true flexibility of the tool.

I have read this submission. I believe that I have an appropriate level of expertise to confirm that it is of an acceptable scientific standard.

# Response

**Published:** 2014-01-24

**Authors:** Robert Davey

**Affiliations:** The Genome Analysis Centre, UK

We would like to thank Mick for taking the time to read and comment on our manuscript.

Admittedly, there is much interplay when considering terminology such as "run" and "analysis", but throughout the paper we use the term "run" to represent a sequencing run, and similarly, the term "analysis" to represent a QC process. We feel this is adequate given the focus of the paper, but we have clarified one potential misuse (changed "run" to "carried out", where appropriate).

Yes, the field refers to these terms regularly, given that "long read" sequencers are available and distinct from their "short-read" counterparts, i.e. we consider the new 2x300bp and upcoming 2x400bp Illumina techniques to still be "short-read".

As long as the data produced from non-Illumina machines is in the FASTQ format (as we state in the existing Use Case text), then there are no differences from the method outlined in the paper, e.g. FASTQC output parsed and stored in StatsDB. Where differences may exist, e.g. STS files from PacBio, different parsers can be written to accommodate this, and we are working on producing such a parser. By no means is StatsDB inherently tied to the FASTQ format, but we feel this reflects the most common QC methods available currently.
